# Cost-effectiveness of virtual patient integration in dermatology education: evidence for precision education by learner expertise

**DOI:** 10.1186/s12909-026-09759-7

**Published:** 2026-07-06

**Authors:** Chih-Tsung Hung, Sheng-Wen Liu, Shou-En Wu, Feng-Cheng Liu

**Affiliations:** 1 Department of Dermatology, National Defense Medical University, Tri- Service General Hospital, Taipei, Taiwan; 2 Department of Dermatology, School of Medicine, College of Medicine, National Defense Medical University, Taipei, Taiwan; 3 Division of Rheumatology/Immunology and Allergy, Department of Internal Medicine, Tri-Service General Hospital, National Defense Medical University, No. 325, Section 2, Chenggong Road, Neihu District, Taipei, 11490 Taiwan

**Keywords:** Virtual patients, Flipped classroom, Cost-effectiveness, Expertise reversal, Precision education, Medical education

## Abstract

**Background:**

Educational technology adoption accelerates despite limited evidence of incremental value within already-effective curricula. Pragmatic evaluations examining cost-effectiveness and differential benefits across learner populations with varying expertise remain scarce.

**Objectives:**

To evaluate virtual patient integration into established flipped classroom curriculum, examining incremental educational outcomes, comprehensive cost-effectiveness, and systematic variation by training level.

**Methods:**

Sequential implementation study at a tertiary teaching hospital in Taiwan (2022–2025). Flipped classroom (FC; 2022–2023, *N* = 131) was followed by FC with virtual patients (FC + VP; 2024–2025, *N* = 161). Primary outcomes: knowledge acquisition (Cohen’s *d*, achievement rates ≥ 80%). Incremental cost-effectiveness ratios (ICERs) were calculated by training level.

**Results:**

Both approaches achieved extremely large learning effects (FC: *d* = 2.27; FC + VP: *d* = 2.43; >98% achievement; between-group differences non-significant). FC + VP required 3.08-fold higher per-student investment ($163 vs. $53). Cost-effectiveness varied dramatically: undergraduates showed meaningful gains (Δ*d* = + 0.29, ICER = $383/*d*), while postgraduates showed minimal benefit (Δ*d* = + 0.02, ICER = $4,670/*d*)—a 12.2-fold difference. Germane cognitive load was associated with learning progress (*r* = .196, *R²* = 8.5%); critical thinking was not independently associated (*p* = .497).

**Conclusions:**

Near-universal competency is attainable through multiple instructional approaches. Virtual patient integration was associated with differential incremental value by learner expertise—meaningful for novices but negligible for advanced learners achieving comparable outcomes more efficiently. Findings support precision education frameworks targeting technology investments based on cost-effectiveness profiles rather than universal deployment.

**Supplementary Information:**

The online version contains supplementary material available at https://doi.org/10.1186/s12909-026-09759-7.

## Introduction

Medical education faces increasing fiscal pressure as educational technology costs escalate while institutional budgets remain constrained [1, 2]. Virtual patients—computer-based simulations of clinical scenarios—represent substantial investment for medical schools, with platform licensing, development, and maintenance costs often exceeding hundreds of thousands of dollars annually [3, 4]. Despite enthusiastic adoption, evidence demonstrating incremental educational value when integrated into already-effective curricula remains limited, and comprehensive cost-effectiveness analyses are scarce [5, 6]. This evidence gap impedes informed resource allocation decisions when medical education requires sustainable innovation strategies.

Flipped classroom approaches, which shift content delivery outside class to dedicate in-class time to active learning, have demonstrated consistent effectiveness in medical education [7, 8]. Recent meta-analyses confirm that flipped classrooms produce superior knowledge acquisition compared to traditional lecture-based instruction, with effect sizes ranging from moderate to large across diverse medical disciplines [9–14].

The pedagogical success of flipped classrooms raises a critical question: does adding expensive virtual patient technology to an already-effective flipped classroom provide sufficient incremental benefit to justify the substantial additional investment? Efficacy trials establishing that virtual patients can improve learning do not address whether they provide meaningful added value beyond simpler, more cost-efficient active learning approaches that may achieve comparable outcomes [15–19]. Critically, no prior study has combined educational effectiveness evaluation with comprehensive cost-effectiveness analysis stratified by learner expertise level, leaving resource allocation decisions without empirical economic guidance.

Cognitive load theory offers a framework for understanding potential differential benefits of enhanced educational scaffolding [20, 21]. The theory predicts that instructional support requirements vary with learner expertise: novice learners benefit from guided instruction reducing cognitive load, while advanced learners with well-developed schemas may find additional guidance redundant—the expertise reversal effect [22, 23]. This theoretical prediction suggests that virtual patient integration might provide differential value, with meaningful benefits for early learners lacking clinical experience but minimal gains for advanced learners who achieve comparable outcomes more efficiently. Despite robust theoretical grounding and experimental support [23–25], the expertise reversal effect has not been systematically examined in authentic medical education contexts with cost-effectiveness analysis.

The precision education concept—adapting instructional approaches to learner characteristics—has gained prominence as medical education adopts competency-based frameworks requiring individualized learning paths [26–29]. However, precision education implementations have focused primarily on pedagogical adaptation without incorporating economic considerations. Understanding not only which learners benefit from enhanced technology but also whether the incremental benefit justifies the incremental investment is essential for sustainable precision education implementation.

This study addresses these gaps by conducting pragmatic evaluation of virtual patient integration into established flipped classroom curriculum in dermatology education. We examined three interrelated questions: (1) Does virtual patient integration provide incremental educational benefit when added to effective flipped classroom? (2) How does cost-effectiveness vary by learner expertise level (undergraduate medical students versus postgraduate residents)? (3) What mechanisms (germane cognitive load, critical thinking) mediate relationships between instructional approach and learning outcomes? By integrating educational effectiveness evaluation with comprehensive cost-effectiveness analysis stratified by training level, this study provides evidence to guide resource allocation decisions and inform precision education frameworks.

## Methods

### Study design

Sequential two-group design comparing flipped classroom (FC) versus FC enhanced with virtual patients (FC + VP) at Department of Dermatology, Tri-Service General Hospital, Taiwan. FC implemented 2022–2023 (baseline), FC + VP 2024–2025. IRB approved (TSGHIRB No. C202105012). Curricular content, faculty, assessment instruments, and rotation structures remained identical across both implementation periods. No major institutional, clinical workflow, or contextual changes occurred during the study period.

### Participants

During 2022–2025, 315 students enrolled: FC period 145 students (93 undergraduate [UGY], 52 postgraduate residents [PGY]); FC + VP period 170 students (84 UGY, 86 PGY). UGY: fifth-year clinical clerkship (2-week rotations). PGY: first- and second-year residents (4-week rotations). Analysis: FC *N* = 131 (85 UGY, 46 PGY, 90.3% completion), FC + VP *N* = 161 (79 UGY, 82 PGY, 94.7% completion), yielding *N* = 292 (92.7% completion, Supplementary Figure S1).

### Interventions

Both targeted identical objectives: competent scabies diagnosis and management [30]. FC: pre-class video lectures (15 min), clinical atlas, readings, quiz; in-class (90 min): review, case discussions, Q&A. FC + VP: identical FC components plus one interactive virtual patient case (30 min total, Virti platform) featuring diverse contexts, interactive examination, diagnostic scaffolds, immediate feedback. All other components constant across periods to isolate virtual patient effects.

### Outcomes

Primary: a 10-item knowledge examination covering four clinical domains: clinical recognition (3 items), diagnostic approach (2 items), treatment decision-making (3 items), and patient education (2 items). The instrument demonstrated good internal consistency (KR-20 = 0.83). Items assessing clinical recognition showed near-ceiling difficulty (*p* > .94), consistent with foundational knowledge expected across training levels. The examination had not been previously validated in external populations; psychometric findings should be interpreted within this single-institution context. Measures: learning progress (post-pre), Cohen’s d effect size, achievement rate (≥ 80%). Secondary (FC + VP only): critical thinking disposition (*α* = 0.933), cognitive load [31] (intrinsic *α* = 0.70, extraneous *α* = 0.87, germane *α* = 0.84). CT disposition was assessed using a 5-point Likert scale (1 = *strongly disagree*, 5 = *strongly agree*); cognitive load subscales (ICL, ECL, GCL) were assessed using 7-point Likert scales (1 = *strongly disagree*, 7 = *strongly agree*) per Leppink et al. [31]. Cost-effectiveness: institutional costs (development, operations, technology, materials). Incremental cost-effectiveness ratios (ICERs): [Cost per student (FC + VP) − Cost per student (FC)] / [Cohen’s *d* (FC + VP) − Cohen’s *d* (FC)], computed overall and by training level.

### Statistical analysis

Independent samples t-tests compared baseline knowledge and post-intervention outcomes; paired samples t-tests assessed within-group changes. Chi-square tests compared achievement rates. Pearson correlations examined relationships between process measures and outcomes. Linear regression tested whether process variables predicted progress controlling for training level. *α* = 0.05 (two-tailed). Complete case analysis: 292/315 (92.7%) completed assessments; 23 (7.3%) with incomplete questionnaires excluded (FC: *n* = 14/145; FC + VP: *n* = 9/170). Completion rates were similar across training levels (UGY: 92.7%; PGY: 92.8%; *χ²*(1) = 0.001, *p* = .975). 95% confidence intervals (95% CIs) for Cohen’s *d* were calculated using the standard error approximation *SE* = √(1/*n* + *d*²/2*n*). CIs for Pearson correlation coefficients were derived using Fisher’s *z* transformation.

### Use of artificial intelligence and statistical software

Figure S1 (Participant Flow) was generated using Google Gemini (Gemini 1.5 Pro, Google LLC, Mountain View, CA) based on actual research data collected from this study. The AI tool was used solely for visual presentation and formatting of pre-existing data to enhance clarity and professional appearance; all underlying numerical data were verified against source records to ensure accuracy. Supplementary Figure S2 (Incremental Cost-Effectiveness Ratios by Training Level) was produced using R version 4.4 (R Foundation for Statistical Computing, Vienna, Austria) with the ggplot2 package [32]. All statistical analyses were performed using conventional statistical methods without AI assistance. No AI-generated content was used in the conception, design, data collection, analysis, interpretation, or writing of this manuscript. This use of AI tools complies with Springer Nature author guidelines.

## Results

### Participant characteristics and baseline comparability

Of 315 students (145 FC, 170 FC + VP), 292 (92.7%) completed assessments (Supplementary Figure S1). Final analysis: FC *N* = 131 (85 UGY, 46 PGY), FC + VP *N* = 161 (79 UGY, 82 PGY). Baseline knowledge was comparable between FC and FC + VP groups overall (55.42% vs. 56.02%, *t*(290) = 0.30, *p* = .761) and within training levels (UGY: 55.06% vs. 57.47%, *t*(162) = 0.92, *p* = .361; PGY: 56.09% vs. 54.63%, *t*(126) = 0.46, *p* = .644), supporting baseline comparability (Table 1).

**Table 1:** Baseline characteristics and knowledge assessment

Characteristic	FC	FC + VP	*p*-value
Overall	*N* = 131	*N* = 161	
Pre-test score, mean (SD)	55.42% (17.99)	56.02% (15.94)	0.761
Undergraduate Students (UGY)			
* n*	85	79	
Pre-test score, mean (SD)	55.06% (18.49)	57.47% (14.80)	0.361
Postgraduate Residents (PGY)			
* n*	46	82	
Pre-test score, mean (SD)	56.09% (17.19)	54.63% (16.94)	0.644

*FC*  Flipped Classroom, *FC + VP*  Flipped Classroom with Virtual Patients, *UGY*  Undergraduate medical students (fifth-year clinical clerkship), *PGY*  Postgraduate year 1–2 residents, *SD*  Standard Deviation

*p*-values from independent samples *t*-tests. All *p* > .05, indicating no significant baseline differences between periods

### Learning outcomes and educational effectiveness

Both instructional approaches achieved extremely large learning effects with near-universal competency achievement, consistent with recent evidence on virtual reality and educational technology effectiveness in medical education [15, 16, 33, 34]. In the FC group, undergraduate students demonstrated mean learning progress of 41.18% points (Cohen’s *d* = 2.23, 95% CI [1.83, 2.62], 98.8% achieving ≥ 80% post-test mastery), while postgraduate residents showed mean progress of 40.43% points (*d* = 2.35, 95% CI [1.78, 2.93], 97.8% achievement). In the FC + VP group, undergraduates progressed 37.22% points (*d* = 2.51, 95% CI [2.06, 2.96], 100% achievement) and postgraduates progressed 40.24% points (*d* = 2.38, 95% CI [1.95, 2.80], 97.6% achievement) (Table 2). All within-group pre-post comparisons reached strong statistical significance (all *p* < .001), reflecting substantial and robust learning gains across all conditions and training levels. Substantial ceiling effects were evident, with 73–85% of participants achieving perfect post-test scores, suggesting assessments captured learning to competency but potentially lacked discrimination at highest performance levels.

**Table 2:** Learning outcomes by training level

**Outcome**	**FC**	**FC+VP**	***p*** **-value**
Undergraduate Students (UGY)			
Pre-test score, %	55.06	57.47	.361
Post-test score, %	96.24	94.68	.283
Learning progress, %	41.18	37.22	—
Cohen's *d *(95% CI)	2.23 (1.83, 2.62)	2.51 (2.06, 2.96)	—
Incremental Δ*d*	—	+0.29	—
Achievement rate (≥ 80%), %	98.8	100.0	1.000
Postgraduate Residents (PGY)			
Pre-test score, %	56.09	54.63	.644
Post-test score, %	96.52	94.88	.348
Learning progress, %	40.43	40.24	—
Cohen's *d *(95% CI)	2.35 (1.78, 2.93)	2.38 (1.95, 2.80)	—
Incremental Δ*d*	—	+0.02	—
Achievement rate (≥ 80%), %	97.8	97.6	1.000
Overall			
Post-test score, %	96.34	94.78	.157
Achievement rate (≥ 80%), %	98.5	98.8	.748
Cohen's *d *(95% CI)	2.27 (1.94, 2.60)	2.43 (2.12, 2.74)	—
Incremental Δ*d*	—	+0.16	—

*FC* Flipped Classroom, *FC+VP* Flipped Classroom with Virtual Patients, Δ*d* incremental effect size (FC+VP minus FC)

Achievement rate = percentage scoring ≥ 80% on post-test (institutional mastery threshold). Cohen's *d* calculated as mean learning progress divided by pre-test standard deviation. 95% CI = 95% confidence interval for Cohen's *d*. All within-group pre-post comparisons significant at *p* < .001 (paired *t*-tests). Between-group *p*-values from independent samples *t*-tests (continuous outcomes) or *χ*² tests (achievement rates)

Between-group comparisons revealed no statistically significant differences in post-test performance or achievement rates. Overall post-test scores were statistically equivalent (FC: 96.34% vs. FC + VP: 94.78%, *t*(290) = 1.42, *p* = .157). Achievement rates exceeded 98% in both groups (FC: 98.5% vs. FC + VP: 98.8%, *χ²*(1) = 0.10, *p* = .748). Within specific training levels, no significant differences emerged for any outcome measure (UGY post-test: *t*(162) = 1.08, *p* = .283; PGY post-test: *t*(126) = 0.94, *p* = .348; UGY achievement: *χ²*(1) = 0.94, *p* = 1.000; PGY achievement: *χ²*(1) = 0.01, *p* = 1.000). While FC + VP groups demonstrated numerically larger effect sizes compared to FC groups (overall: Δ*d* = + 0.16; UGY: Δ*d* = + 0.29; PGY: Δ*d* = + 0.02), these incremental differences did not reach conventional statistical significance thresholds, indicating educational equivalence between instructional approaches on primary knowledge acquisition outcomes (Table 2).

Exploratory baseline knowledge stratification analysis suggested patterns of differential response warranting future investigation. Among learners entering with low baseline knowledge (≤ 40% pre-test scores), both approaches achieved remarkably large effect sizes (FC: *d* = 7.07, 95% CI [5.61, 8.53]; FC + VP: *d* = 6.93, 95% CI [5.51, 8.35], Δ*d* = − 0.14), with FC achieving 95.9% mastery and FC + VP achieving 100% mastery, though this difference did not reach statistical significance (*χ²*(1) = 1.52, *p* = .466). Among learners with medium baseline knowledge (40–60% pre-test), both approaches achieved 100% mastery in FC and 97.6% in FC + VP (*χ²*(1) = 2.08, *p* = .692). Among learners with high baseline knowledge (> 60% pre-test), both approaches achieved 100% mastery rates. The inability to calculate meaningful effect sizes for medium and high baseline strata (*d* = 0.00 due to ceiling effects) suggests potential ceiling constraints on observable differential effectiveness at higher baseline knowledge levels (Supplementary Table S1).

### Process measures: cognitive load and critical thinking

Within the FC + VP group, germane cognitive load—representing learning-directed cognitive processing—showed a small positive correlation with learning progress (*r* = .196, 95% CI [0.042, 0.340], *p* = .013), while extraneous cognitive load showed a small negative correlation (*r* = − .241, 95% CI [− 0.382, − 0.090], *p* = .002). Critical thinking disposition showed no significant bivariate correlation with learning progress (*r* = .015, 95% CI [− 0.140, 0.169], *p* = .854). Multiple linear regression controlling for training level and all process measures simultaneously indicated that no single process variable reached conventional significance thresholds: germane cognitive load showed the largest positive association (*β* = 3.39, *SE* = 1.78, *p* = .059), while critical thinking was not associated with learning progress (*β* = −1.89, *SE* = 2.78, *p* = .497), indicating that approximately 8.5% of variance in learning outcomes was attributable to cognitive load factors collectively (*R*² = 0.085; Supplementary Table S2). The limited explained variance reflects the near-universal competency ceiling achieved by both groups and the multifactorial complexity of learning outcomes in authentic educational contexts.

### Cost-effectiveness analysis

Annual operational costs differed substantially between instructional approaches. The FC approach required $3,460 annually serving 66 students ($53 per student), while the FC + VP approach required $13,108 annually serving 80 students ($163 per student), representing a 3.08-fold higher per-student investment associated with virtual patient integration.

Cost-effectiveness analysis revealed dramatic variation by training level (Supplementary Figure S2). For undergraduate medical students, virtual patient integration demonstrated favorable cost-effectiveness with an incremental cost-effectiveness ratio (ICER) of $383 per Cohen’s *d* unit, reflecting meaningful effect size gains (Δ*d* = + 0.29) coupled with achievement of universal mastery (100% vs. 98.8%). In striking contrast, for postgraduate residents the ICER was $4,670 per Cohen’s *d* unit, reflecting minimal incremental benefit (Δ*d* = + 0.02) with no observable change in achievement rates (both groups 97.6–97.8%). This represents a 12.2-fold difference in cost-effectiveness between training levels, with virtual patient integration being associated with substantially superior value for novice learners compared to advanced learners who achieved comparable educational outcomes more efficiently through the less resource-intensive flipped classroom approach (Table 3).

**Table 3:** Cost-effectiveness analysis by training level

Component	FC	FC + VP	Incremental
Annual Operational Costs			
Total annual cost	$3,460	$13,108	+$9,648
Students served per year	66	80	—
Cost per student	$53	$163	+$110 (3.08-fold)
Overall Cost-Effectiveness			
Cohen’s *d*	2.27	2.43	+ 0.16
ICER ($ per *d* unit)	—	—	$703
Undergraduate Students (UGY)			
Cohen’s *d*	2.23	2.51	+ 0.29
Achievement rate	98.8%	100.0%	+ 1.2%
ICER ($ per *d* unit)	—	—	$383
Postgraduate Residents (PGY)			
Cohen’s *d*	2.35	2.38	+ 0.02
Achievement rate	97.8%	97.6%	−0.3%
ICER ($ per *d* unit)	—	—	$4,670
Cost-Effectiveness Differential			
UGY vs. PGY ICER ratio	—	—	12.2-fold

*FC* Flipped Classroom, *FC+VP* Flipped Classroom with Virtual Patients, *ICER* Incremental Cost-Effectiveness Ratio, calculated as: [Cost per student (FC+VP) − Cost per student (FC)] / [Cohen's *d* (FC+VP) − Cohen's *d* (FC)], representing additional cost per unit increase in effect size

## Discussion

This pragmatic evaluation reveals that both flipped classroom alone (FC) and flipped classroom with virtual patients (FC + VP) achieved extremely large learning effects (Cohen’s *d* > 2.2) with near-universal competency achievement (> 98%), demonstrating educational equivalence on primary knowledge acquisition outcomes. However, comprehensive cost-effectiveness analysis revealed dramatic differential value by learner expertise: virtual patient integration was associated with meaningful incremental gains for undergraduate students (Δ*d* = + 0.29, universal achievement, ICER = $383 per Cohen’s *d* unit) but negligible gains for postgraduate residents (Δ*d* = + 0.02, ICER = $4,670 per Cohen’s *d* unit)—a striking 12.2-fold difference in cost-effectiveness (Supplementary Figure S2). Germane cognitive load showed moderate correlation with learning progress (*r* = .196, *R*² = 8.5% for cognitive load factors collectively), while critical thinking showed no significant bivariate correlation (*r* = .015) and no independent predictive value in multivariate analysis. These findings collectively support precision education frameworks wherein technology investments are strategically targeted based on demonstrated cost-effectiveness profiles and learner expertise characteristics.

The extremely large effect sizes in both groups align with meta-analytic evidence demonstrating flipped classroom superiority over traditional instruction across medical disciplines [7–12, 14]. Near-universal achievement rates (> 98%) indicate that well-designed active learning environments can successfully bring learners to competency regardless of technological enhancement, with important implications for competency-based medical education [35].

The observed pattern—meaningful gains for undergraduates but minimal for postgraduates—is consistent with cognitive load theory’s expertise reversal effect [22, 24]. A recent meta-analysis of 60 studies confirms that low prior knowledge learners benefit from high-assistance instruction (*d* = 0.505) while high prior knowledge learners benefit from low-assistance instruction (*d* = − 0.428) [23]. For undergraduate students lacking dermatological clinical exposure, virtual patients may have provided structured scaffolding associated with reduced extraneous load while promoting germane load through realistic case engagement. For postgraduate residents with developed clinical schemas from patient care rotations, virtual patient scaffolding likely introduced redundancy relative to internal knowledge structures, potentially increasing cognitive load—the hallmark of expertise reversal. It should be noted, however, that the near-universal achievement ceiling for postgraduates (97.6–97.8%) may have limited the measurable range of any incremental gains. The 12.2-fold ICER difference suggests that targeted deployment may be not merely pedagogically sound but also economically important for sustainable implementation [3, 4], though this conclusion should be interpreted within the context of the assessment sensitivity constraints described in the Limitations section.

Germane cognitive load’s small positive correlation with learning progress (*r* = .196, explaining approximately 8.5% of variance when considering all cognitive load factors collectively) is directionally consistent with cognitive load theory predictions [20, 21, 31], while reflecting real-world educational complexity where multiple factors influence outcomes. Critical thinking disposition showed no significant bivariate or multivariate association with learning progress (*r* = .015; *β* = −1.89, *p* = .497) in multivariate analysis, suggesting that general cognitive dispositions may not directly drive knowledge acquisition in focused competency-based interventions, consistent with domain-specificity principles [16].

Several important limitations warrant consideration. First, sequential implementation raises concerns about temporal confounding and history bias. However, multiple factors mitigate this concern: baseline knowledge equivalence (*p* = .761), consistent instructor and curriculum across periods, identical assessments, and stable institutional context. The extremely large effect sizes in both periods (*d* > 2.2) suggest robust effectiveness independent of temporal factors. Moreover, the observed pattern—meaningful gains for undergraduates but minimal for postgraduates—is consistent with expertise reversal theory predictions [22, 23] rather than reflecting secular trends, as temporal effects would be expected to influence both groups similarly. While randomized concurrent designs would provide stronger causal inference, the convergent evidence from baseline equivalence, theoretical alignment, and differential response patterns by expertise level supports validity of cost-effectiveness conclusions.

Second, substantial ceiling effects present critical measurement limitations. Postgraduate residents achieved near-universal competency (FC: 97.8% vs. FC + VP: 97.6%, *p* = 1.000) with minimal incremental effect associated with virtual patient integration (Δ*d* = + 0.02), while 77–85% attained perfect post-test scores. This strongly suggests the assessment tool lacked sufficient difficulty to discriminate learning gains at high performance levels. The apparent lack of incremental benefit for postgraduates should be interpreted cautiously—it may partially reflect assessment insensitivity rather than true absence of learning gains. Future research should employ assessments with greater difficulty and discrimination at high performance levels—incorporating complex clinical scenarios, atypical presentations, or management of complications—to detect potential subtle benefits current measures cannot capture.

However, this measurement limitation paradoxically strengthens rather than weakens cost-effectiveness conclusions from a pragmatic perspective. Even assuming unmeasured incremental benefits exist for postgraduates, three converging factors support conclusions: (1) both approaches successfully achieved bringing learners to competency (> 97% achievement rates); (2) the 3.08-fold higher per-student investment ($163 vs. $53) represents substantial additional resource commitment; (3) any unmeasured benefits would necessarily be marginal improvements beyond already-near-perfect performance. The critical question becomes whether potential marginal improvements beyond near-universal competency achievement justify substantial additional investment, particularly when simpler approaches achieve comparable competency rates more efficiently.

Third, this study focused on scabies management—a relatively circumscribed dermatological condition. Generalizability to more complex scenarios requires empirical verification. However, scabies was deliberately selected for several compelling reasons: it represents essential foundational knowledge encountered across all medical specialties; it requires integrating clinical recognition with diagnostic procedures; it demands authentic clinical reasoning including pattern recognition across varying presentations (classic to crusted), evidence-based treatment decision-making considering patient-specific factors, and patient education regarding transmission and compliance. Focusing on a single well-defined condition with identical learning objectives provided experimental control for rigorous cost-effectiveness comparison isolating virtual patient effects. The observed expertise reversal pattern reflects fundamental cognitive load principles likely to generalize across content domains. Nevertheless, future research should examine whether cost-effectiveness differentials persist across more complex dermatological conditions and other medical domains.

Additional limitations include that process measures were assessed only in the FC + VP group, preventing direct mechanistic comparison between approaches; findings derive from one institution requiring cross-cultural and cross-institutional validation; and cost-effectiveness analysis captured direct educational costs without accounting for indirect costs (opportunity costs, long-term retention effects) or broader value considerations (learner satisfaction, clinical performance outcomes).

These findings offer practical guidance for educational leaders. First, excellent outcomes are achievable through multiple pedagogical approaches; pedagogical principles matter more than technological sophistication for foundational knowledge acquisition. Second, technology investments should be targeted rather than universal. The 12.2-fold ICER difference indicates indiscriminate deployment is economically inefficient. Institutions should prioritize enhanced technologies for learners with demonstrated need while employing simpler approaches for advanced learners achieving comparable outcomes efficiently. Third, comprehensive evaluation frameworks integrating educational effectiveness with cost-effectiveness analysis are essential for informed decision-making [3, 36, 37], as pedagogical equivalence may mask substantial economic disparities with important implications for sustainable resource allocation.

In conclusion, this pragmatic evaluation suggests that while flipped classroom approaches achieve near-universal competency, the incremental value of virtual patient integration appears to vary dramatically by learner expertise. Findings support precision education frameworks wherein technology investments are strategically targeted based on cost-effectiveness profiles rather than deployed universally. As medical education adopts competency-based models requiring individualized learning paths [29, 35], understanding the economic sustainability of pedagogical approaches becomes as essential as evaluating educational effectiveness, particularly as artificial intelligence and digital technologies continue to transform medical education delivery and create new opportunities for precision education implementation [38–41]. Future research should examine whether these cost-effectiveness differentials persist across more complex clinical conditions and diverse institutional contexts, and employ randomized concurrent designs to strengthen causal inference.

## Supplementary Information

Supplementary Material 1.

## Data Availability

The datasets generated and/or analysed during the current study are not publicly available due to institutional data governance requirements and participant privacy considerations, but are available from the corresponding author on reasonable request.
