# Supplementary material for: Cost-effectiveness of virtual patient integration in dermatology education: evidence for precision education by learner expertise
Source: BMC Med Educ. 2026 Jul 6;26:1541. doi: 10.1186/s12909-026-09759-7 (PMC13625295; doi:10.1186/s12909-026-09759-7)
Supplement: Supplementary file 1 [file 12909_2026_9759_MOESM1_ESM.docx]

**Supplementary Materials**

*Cost-Effectiveness of Virtual Patient Integration in Dermatology Education:*

*Evidence for Precision Education by Learner Expertise*

**Contents**

Supplementary Table S1. Learning Outcomes Stratified by Baseline Knowledge Level

Supplementary Table S2. Process Measures: Cognitive Load and Critical Thinking (FC+VP Period, N = 161)

Supplementary Figure S1. Participant Flow Diagram

Supplementary Figure S2. Incremental Cost-Effectiveness Ratio (ICER) by Training Level

**Supplementary Table S1.** Learning Outcomes Stratified by Baseline Knowledge Level

| **Baseline Knowledge Level** | **Measure** | **FC** | **FC+VP** | ***p*-value** |
| --- | --- | --- | --- | --- |
| **Low (≤ 40%)** | *n* | 49 | 50 |  |
|  | Cohen's *d* (95% CI) | 7.07 (5.61, 8.53) | 6.93 (5.51, 8.35) |  |
|  | Incremental Δ*d* | — | −0.14 |  |
|  | Achievement rate (%) | 95.9 | 100.0 | .466 |
| **Medium (40–60%)** | *n* | 52 | 83 |  |
|  | Cohen's *d* (95% CI) | 0.00ᵃ | 0.00ᵃ |  |
|  | Incremental Δ*d* | — | 0.00 |  |
|  | Achievement rate (%) | 100.0 | 97.6 | .692 |
| **High (> 60%)** | *n* | 30 | 28 |  |
|  | Cohen's *d* (95% CI) | 0.00ᵃ | 0.00ᵃ |  |
|  | Incremental Δ*d* | — | 0.00 |  |
|  | Achievement rate (%) | 100.0 | 100.0 | 1.000 |

*Note.* FC = Flipped Classroom; FC+VP = Flipped Classroom with Virtual Patients; Δ*d* = incremental effect size (FC+VP minus FC). ᵃCohen's *d* = 0.00 due to ceiling effects—in medium and high baseline strata, all or nearly all students achieved maximum or near-maximum post-test scores, limiting score variability needed to calculate meaningful effect sizes.

**Supplementary Table S2.** Process Measures: Cognitive Load and Critical Thinking (FC+VP Period, *N* = 161)

| **Measure** | **Items** | **Mean (SD)** | **Cronbach α** | ***r* with Progress** | **Regression *β* (*SE*)** | ***p*-value** |
| --- | --- | --- | --- | --- | --- | --- |
| Critical Thinking Disposition (CT) | 6 | 4.56 (0.50) | .933 | .015 | −1.89 (2.78) | .497 |
| Intrinsic Cognitive Load (ICL) | 2 | 4.17 (1.20) | .702 | −.121 | −0.07 (1.46) | .961 |
| Extraneous Cognitive Load (ECL) | 3 | 2.60 (1.19) | .867 | −.241** | −2.66 (1.53) | .084 |
| Germane Cognitive Load (GCL) | 3 | 5.97 (0.82) | .839 | .196* | 3.39 (1.78) | .059 |

*Note.* All measures assessed in FC+VP period only (*N* = 161). CT was assessed using a 5-point Likert scale (1 = *strongly disagree*, 5 = *strongly agree*); ICL, ECL, and GCL were assessed using 7-point Likert scales (1 = *strongly disagree*, 7 = *strongly agree*). Cronbach's α indicates internal consistency reliability (α ≥ .70 considered acceptable). Correlation coefficients (*r*) from Pearson correlation analysis with learning progress (post-test minus pre-test score). Regression coefficients (*β*) and standard errors (*SE*) from multiple linear regression controlling for training level and all four cognitive process measures simultaneously (*R*² = .085). **p* < .05, ***p* < .01, ****p* < .001.

**Supplementary Figure S1.** Participant Flow Diagram.

Sequential implementation study comparing flipped classroom (FC, 2022–2023) versus FC with virtual patients (FC+VP, 2024–2025). FC period: 145 enrolled, 131 completed (90.3% completion rate; *n* = 14 incomplete [UGY = 8, PGY = 6]). FC+VP period: 170 enrolled, 161 completed (94.7% completion rate; *n* = 9 incomplete [UGY = 5, PGY = 4]). Overall: 292/315 (92.7%) completed all assessments. Completion rates did not differ across training levels (UGY: 92.7%; PGY: 92.8%; χ²(1) = 0.001, *p* = .975). Incomplete records defined as failure to complete both pre-test and post-test assessments, or missing questionnaire data; these were excluded from analysis. UGY = undergraduate medical students (fifth-year clinical clerkship, 2-week rotations); PGY = postgraduate year 1–2 residents (4-week rotations).

**Supplementary Figure S2.** Incremental Cost-Effectiveness Ratio (ICER) by Training Level.

Bar chart displaying ICER (USD per unit increase in Cohen's *d* effect size) for virtual patient integration across three comparison groups: overall (*N* = 292), undergraduate students (UGY; *n* = 164), and postgraduate residents (PGY; *n* = 128). FC+VP required $163 per student versus $53 for FC (3.08-fold higher investment; incremental cost = $110 per student). ICER = [FC+VP cost per student − FC cost per student] ÷ [Cohen's *d* (FC+VP) − Cohen's *d* (FC)]. Overall ICER = $703 (Δ*d* = +0.16); UGY ICER = $383 (Δ*d* = +0.29, universal achievement); PGY ICER = $4,670 (Δ*d* = +0.02, no change in achievement rates). The 12.2-fold ICER differential indicates virtual patient integration was associated with substantially more cost-effective incremental benefit for novice undergraduate learners than for advanced postgraduate residents, who achieved comparable educational outcomes through the simpler flipped classroom approach.
